# Natural killer cells in cancer biology and therapy

**DOI:** 10.1186/s12943-020-01238-x

**Published:** 2020-08-06

**Authors:** Song-Yang Wu, Tong Fu, Yi-Zhou Jiang, Zhi-Ming Shao

**Affiliations:** 1 Department of Breast Surgery, Fudan University Shanghai Cancer Center, Shanghai, 200032 China; 2 Department of Oncology, Key Laboratory of Breast Cancer in Shanghai, Shanghai Medical College, Fudan University, Shanghai, 200032 China

**Keywords:** Innate immunity, Natural killer cell, Tumor progression, Immunometabolism, Immunotherapy, Precision treatment

## Abstract

The tumor microenvironment is highly complex, and immune escape is currently considered an important hallmark of cancer, largely contributing to tumor progression and metastasis. Named for their capability of killing target cells autonomously, natural killer (NK) cells serve as the main effector cells toward cancer in innate immunity and are highly heterogeneous in the microenvironment. Most current treatment options harnessing the tumor microenvironment focus on T cell-immunity, either by promoting activating signals or suppressing inhibitory ones. The limited success achieved by T cell immunotherapy highlights the importance of developing new-generation immunotherapeutics, for example utilizing previously ignored NK cells. Although tumors also evolve to resist NK cell-induced cytotoxicity, cytokine supplement, blockade of suppressive molecules and genetic engineering of NK cells may overcome such resistance with great promise in both solid and hematological malignancies. In this review, we summarized the fundamental characteristics and recent advances of NK cells within tumor immunometabolic microenvironment, and discussed potential application and limitations of emerging NK cell-based therapeutic strategies in the era of presicion medicine.

## Background

The diversity of infiltrating stromal cells occurring in human cancers exceeds 30 distinct subgroups, reflecting the huge complexity of the tumor microenvironment (TME), thereby deeply affecting the treatment option for each patient [1]. Attempts have been made to distill this out-of-order situation into a unifying method to better describe actual composition of the TME using both multi-omics and experimental technologies, shedding light on cancer biology. This trend led to a transition in cancer treatment from only targeting tumor cells (like traditional chemotherapy and radiotherapy) to a new generation of approaches emphasizing the modulation of endogenous immune response toward cancer.

The immune system can be generally divided into the innate and adaptive immune systems, both contributing to the recognition and removal of foreign pathogens as well as tumors [2]. Adaptive immunity is mainly composed of cells represented by T and B lymphocytes, which harbor an enormous repertoire of T-cell and B-cell receptors, respectively, that can respond specifically to different antigens in the body. Current immunotherapeutic methods mainly focus on T lymphocytes, especially restoring exhausted CD8^+^ cytotoxic T cells (CTLs). An example of such approach is immune-checkpoint blockade, with blocking of receptors or ligands that inhibit the activation of CTLs, including programmed cell death protein 1 (PD-1), its main ligand PD-L1, cytotoxic T-lymphocyte antigen 4 (CTLA-4) and lymphocyte-activation gene-3 (LAG-3), by monoclonal neutralizing antibodies [3, 4]. In recent years, the rapid and potent anti-tumor function of innate immunity, which even occurs at a very early stage of tumor progression, has attracted increasing attention. As a subset of whole innate lymphoid cells, natural killer (NK) cells, defined by Herberman in 1976 [5] and often considered a part of type 1 innate-like cells (ILC1s), are currently defined as effector cells similar to CTLs, exerting natural cytotoxicity against primary tumor cells and metastasis by inhibiting proliferation, migration and colonization to distant tissues [6]. Beside their cytotoxic role, NK cells have been reported to produce a large number of cytokines, mainly interferon-γ (IFN-γ), to modulate adaptive immune responses and participate in other related pathways [7, 8]. In addition, as documented in multiple models and experiments, NK cells could distinguish abnormal cells from healthy ones, leading to more specific anti-tumor cytotoxicity and reduced off-target complications [9, 10].

Considering the pivotal role of NK cells in cancer biology, they naturally emerged as a prospective target for cancer therapy, and a growing number of studies and multiple therapeutic agents inhibiting cancer target NK cell-related pathways. In this review, we will review the fundamental characteristics and emerging subpopulations of NK cells. Next, we will mainly use breast cancer (BC) to discuss the plasticity of NK cells in cancer biology and metabolism, as well as current therapeutic regimens, including ongoing clinical trials and FDA-approved therapies targeting NK cells, and future possible approaches for improving cancer treatment.

### Development of NK cells

**Fig. 1:**
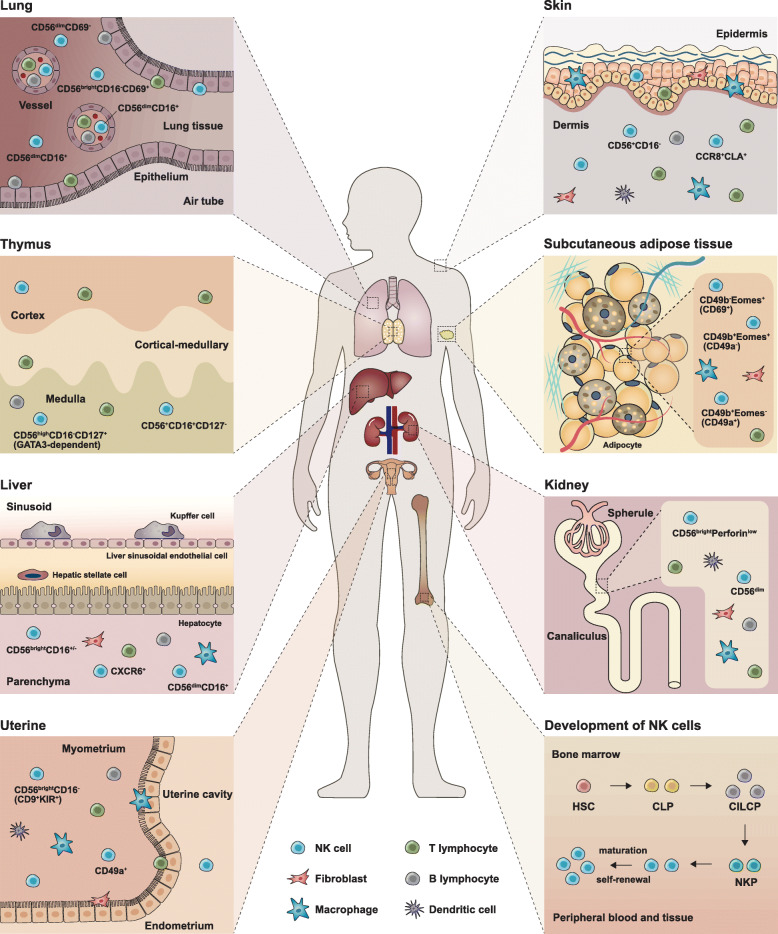
Development and subgroups of NK cells. In bone marrow, NK cells develop from hematopoietic stem cells (HSCs) through common lymphoid progenitors (CLPs) and NK cell precursors (NKPs), and then migrate to peripheral blood (cNK cells) or tissue (trNK cells). The differentiation of trNK-cells occurs in distinct tissue sites, including the lung, thymus, liver, uterus, skin, subcutaneous adipose tissue, and kidney. In these sites, NK cells have different phenotypic features and functions, which constitute the circulation of NK cells at different stages of maturation. CLA, cutaneous lymphocyte-associated antigen; CCR8, C-C motif chemokine receptor 8; GATA3, GATA binding protein 3; CXCR6, C-X-C motif chemokine receptor 6; KIR, killer cell immunoglobulin-like receptor; CILCP, common innate-like cell precursor

NK cells possess cytotoxic abilities similar to CD8^+^ T cells functioning in the adaptive immunity but lack CD3 and the T cell receptors (TCRs). Largely circulating in blood and counting for about 5–10% of peripheral blood mononuclear cells (PBMCs), NK cells are found in bone marrow and lymphoid tissues such as the spleen [11, 12]. Similar to other ILCs, NK cells are originated from common lymphoid progenitor (CLP) cells in bone marrow (Fig. 1) with an average renewal cycle of about 2 weeks [12]. During development, a process termed education, which describes the interaction of NK cells expressing immunoreceptor tyrosine-based inhibitory motifs (ITIMs) with major histocompatibility complex-I (MHC-I), helps NK cells become licensed and avoid attacking healthy normal cells [6, 9]. Interestingly, tumor cells always lack or only express low levels of MHC-I to evade CD8^+^ T cell-mediated cytotoxicity, whereas licensed NK cells are fully activated. However, tumor cells also express molecules that activate NK cells, e.g., MHC class I polypeptide-related sequence A (MICA) and MICB [13, 14], supporting the use of NK cells as anti-cancer agents. In addition, unlicensed NK cells also play important roles in the body, e.g., eliminating murine cytomegalovirus (MCMV) infection and MHC-I^+^ cells [15].

To date, NK cell survival and development is thought to mainly rely on cytokines (especially IL-2 and IL-15) [16–19] and transcription factors (Nfil3, Id2 and Tox for development, and EOMES and T-bet for maturation) [16, 20]. GRB2-associated binding protein 3 (GAB3) is essential for IL-2 and IL-15-mediated, and its deficiency leads to impaired NK cell expansion [21]. In addition, targeting related signals is a potential option for promoting NK cell-induced cytotoxicity toward cancer. As reported previously, ablation of cytokine-inducible SH2-containg protein (CIS), which negatively regulates IL-15 to restrict NK cell function, could prevent metastasis and also potentiate CTLA-4 and PD-1 blockade therapy in vivo [22].

### Identification and molecular features of NK cells

#### Surface molecules of NK cells

Due to variable expression of surface markers on NK cells, it is hard to use one or two simple molecules or traditional immunohistochemistry to accurately identify this cell type and more importantly, their functional status. However, in humans, in both clinical and research settings, CD3^−^CD56^+^ cells are commonly identified as NK cells and can be further divided into the CD56^bright^ and CD56^dim^ subgroups. CD56 is not only a marker but also plays an important role in the terminal differentiation of NK cells since its blockade by monoclonal antibodies obviously inhibits the transition from CD56^bright^ to CD56^dim^, thus limiting the cytotoxic ability [23]. Consistently, CD3^−^NK1.1^+^ and CD3^−^CD49b^+^ cells are defined as NK cells in mice. In recent studies, the notion that natural cytotoxicity receptor 46 (NKp46), belonging to natural cytotoxicity receptors (NCRs), should also be included in this panel has been proposed based on the consensus of adding more functional proteins rather than surface molecules into the classification system of NK cells [24, 25].

#### Activating and inhibitory signals in NK cells

As the main effector cell type in innate immunity, NK cells are capable of killing tumor cells and virus-infected cells at a very early stage. Due to the lack of abundant production of receptors for distinguishing incalculable antigens in the body specifically, they rely on the “missing self” and “induced self” modes to identify target cells by maintaining a precise balance between activating co-stimulatory and inhibitory signals (mainly by functional receptors). Those interacting signals finally decide the activation and functional status of NK cells.

Activating signals include cytokine-binding receptors, integrins, killing-receptors (CD16, NKp40, NKp30 and NKp44), receptors recognizing non-self-antigens (Ly49H) and other receptors (e.g., NKp80, SLAMs, CD18, CD2 and TLR3/9) [26, 27]. In total, the activating receptors of NK cells can be divided into at least three types according to the respective ligands, including MHC-I specific, MHC-I related and MHC-I non-related receptors (Table 1) [13, 28–43]. To emphasize, NCRs, which belong to the third group, include three molecules (NKp30, NKp44 and NKp46), and NKp30 was shown to be capable of recognizing B7-H6 expressed on tumor cells, and could be used as a novel treatment option in the future [35].

**Table 1:** Key mediators of NK cells

Classification	Mediator	Host ligand	Ref.
**NK cell activator**			
MHC-I specific receptor	KIR2DS1, 2DS3, 3DS5	MHC I	[28]
	Ly49c, Ly49i	MHC I	[29]
	NKp80	MHC I	[30]
	NKG2C, NKG2E	MHC I	[31]
MHC-I related receptor	NKG2D	MICA, MICB, ULBPs	[13]
MHC-I non-related receptor	DNAM1	Nectin-2, CD155	[32]
	NKp46 (NCR1)	HS GAGs, CFP	[33]
	NKp44 (NCR2)	HS GAGs, MLL5, NKp44L, PCNA, BAT3, PDGF-DD, Nidogen-1	[34]
	NKp30 (NCR3)	HS GAGs, B7-H6, Galectin-3	[35]
	Nkp65	Keratinocyte-associated C-type lectin	[36]
	LFA-1 (αLβ2 integrin)	Intercellular cell adhesion molecule 1	[37, 38]
	α4 integrin	Vascular cell adhesion molecule 1	[39]
	CD16	Fc-γ	[40, 41]
	CD2	CD581	[41]
	TLR3	Microbial constituents, adjuvant	[42]
	TLR9	CpG	[43]
**NK cell inhibitor**			
MHC-I specific receptor	KIR3DL1	MHC I	[44]
	KIR2DL3, 2DL1	MHC I	[45]
	NKG2A	MHC I	[46]
	KLRB1, LLT1	MHC I	[47]
	LILRB1, LILRB2	MHC I	[48]
MHC-I non-related receptor	KLRG1	E-, N-, and R- cadherins	[49]
	siglec-3, siglec-7, siglec-9	Sialic acid	[50, 51]
	CEACAM1	CEACAM1, CEACAM5	[52]
	2B4 (CD244)	CD48	[53]
	IRp60	Phosphatidylserine	[54]
	LAIR1	Ep-CAM	[55]
	CD96	CD155	[56]
	CD73	Antibodies	[57]
	PD-1	PD-L1	[58]
	TIGIT	CD155	[20]
	NKR-P1B	Clr-b	[59]
	LAG3	MHC-II	[60]

*Abbreviations: MHC* major histocompatibility complex, *KIR* killer cell immunoglobulin-like receptor, *MIC* MHC class I chain-related, *ULBP* UL16-binding protein 1, *DNAM1* DNAX accessory molecule 1, *NCR* natural cytotoxicity receptor, *HS GAGs* heparan sulfate glycosaminoglycans, *CFP* complement factor P, *MLL5* mixed-lineage leukemia protein-5, *PCNA* proliferating cell nuclear antigen, *BAT3* HLA-B-associated transcript 3, *PDGF-DD* platelet-derived growth factor-DD, *LFA-1* lymphocyte function-associated antigen-1, *TLR* toll-like receptor, *KLR* killer cell lectin-like receptor, *LLT1* lectin-like transcript 1, *LILR* leukocyte immunoglobulin-like receptor, *Siglec* sialic acid-binding immunoglobulin-like lectin, *CEACAM* carcinoembryonic antigen-related cell-adhesion molecule, *IRp60* inhibitory receptor protein 60, *LAIR1* leukocyte-associated immunoglobulin-like receptor 1, *Ep-CAM* epithelial cellular adhesion molecule, *PD-1* programmed cell death protein 1, *TIGIT* T-cell immunoreceptor with Ig and ITIM domains, *LAG3* lymphocyte activation gene 3

Inhibitory signals mainly comprise receptors recognizing MHC-I, such as Ly49s, NKG2A and LLT1, as well as some MHC-I non-related receptors (Table 1) [20, 44–60]. Moreover, MHC-I specific inhibitory receptors can be generally divided into three types according to structure and function: killer cell immunoglobulin-like receptors (KIRs), killer lectin-like receptors (KLRs) and leukocyte immunoglobulin-like receptors (LILRs).

#### NK cell subpopulations according to site of maturation

Conventional NK (cNK) cells are mainly found in peripheral blood and migrate to a specific location to exert their effects. NK cells also include tissue-resident NK (trNK) cells. The complex process of NK-cell differentiation occurs in several distinct tissues, including bone marrow, liver, thymus, spleen and lymph nodes, and may involve cell circulation at different stages of maturation among these tissues [61]. In bone marrow, blood, spleen and lungs, NK cells are fully differentiated, while that in lymph nodes and intestines are immature and precursory [62]. Single-cell transcriptome ananlysis of bone marrow and blood NK cells helps to illustrate the changes of their characteristics during development. For example, high expreesion of TIM-3, CX3CR1 and ZEB2 represents a more mature status [63]. Many hypotheses have been proposed to describe the motivation of their migration and different biological behaviors of identically originated NK cells in different tissues. The first question could be partly explained by the multi-direction differentiation induced by heterogeneous microenvironments in different tissues, or more straightforward, different phenotypes originated from similar chemokine-recruited peripheral cNK cells.

To conclude, NK cells in various tissues have diverse features, possessing different functions and forming a close relationship with other stromal cells (Fig. 1). In the lung, trNK cells show a different phenotype from that of circulating NK cells (mainly CD56^dim^) and are considered to express different levels of CD16, CD49a and CD69, with CD56^dim^CD16^+^ cells representing the majority of the whole NK family there [64, 65]. Of note, CD69^+^ cells are the main type of CD56^bright^CD16^−^ NK cells. However, in the thymus, most NK cells are CD56^high^CD16^−^CD127^+^, highly relying on GATA3 compared with the CD56^+^CD16^+^ subgroup [66]. Besides, they produce more effector molecules, including TNF-α and IFN-γ [66, 67]. Similar to the phenotypic features in humans, skin NK cells in the mouse can be generally divided into two types: CD49a^+^DX5^−^ and CD49a^−^DX5^+^ [68, 69]. Similarly, hepatic trNK cells can be classified into two groups, including CD56^bright^CD16^+/−^ and CD56^dim^CD16^+^, both lacking CD3 and CD19 [8]. In addition, CD49a^+^CD56^+^CD3^−^CD19^−^ NK cells have been identified in liver biopsies [70]. Besides, hepatic NK cells can develop memory for structurally diverse antigens, dependent on the surface molecule CXCR6 [71]. In the uterus, most NK cells are CD56^bright^CD16^−^, expressing high levels of KIRs [72]. Decidual NK cells are also CD49a^+^. For skin NK cells, it is intriguing that only few CD56^+^CD16^+^ can be detected, which are common in peripheral blood [73]. Interestingly, trNK cells are distinct between subcutaneous (CD56^dim^) and visceral (CD56^bright^) adipose tissues, and can be generally divided into three groups according to CD49b and Eomes, showing different expression levels of CD49a (CD49b^+^Eomes^−^ subgroup) and CD69 (CD49b^−^Eomes^+^ subgroup) [74, 75].

Besides different tissue types, NK cells are also highly heterogeneous even in the same organ and the same tissue [61]. Through high-dimensional single-cell RNA-seq, Crinier et al. revealed the heterogeneity of human and mouse NK cells in spleen and blood and identified several subpopulations of NK cells, respectively [76]. As mentioned above, NK cells are considered a subgroup of ILC1s [6]. Although ILC1s are not detectable in many tissues, intra-epithelial ILC1 (ieILC1)-like cells, which highly express IFN-γ, integrins and other cytotoxic molecules similar to the ieILC1s previously described by Fuchs except for different NKp44 expression, could represent the majority of NK cells in the mucosal tissue [61, 77]. Due to their unique features, this cell type represents a subgroup of NK cells other than conventional ILCs. Unlike other trNK cells, DX5^−^CD11c^hi^ liver-resident NK cells participate in autoimmune cholangitis, negatively regulating immune responses, especially by inhibiting the proliferation and function of CD4^+^ T cells in vivo, which was validated by severer biliary disease in NK-depleted mice resulting from Nfil3 knockdown or treatment with neutralizing antibodies [78].

#### NK cell subpopulations according to functional molecules

According to surface CD56 expression, NK cells can be divided into CD56^bright^ and CD56^dim^. CD56^dim^ NK cells are mainly found in peripheral blood, and are always also CD16-positive, expressing high levels of KIR and LFA-1 and showing cell killing ability. CD16 is a key receptor mediating antibody-dependent cell cytotoxicity (ADCC), inducing the phosphorylation of immunoreceptor tyrosine-based activation motif (ITAM) [40, 79, 80]. According to a time-resolved single-cell assay, the cytotoxicity of NK cells is repressed through both necrosis and apoptosis. As a result, FasL/FasR interaction, perforin/granzyme release and Ca2^+^ influx are all important for NK cell function [81]. However, CD56^bright^ NK cells are similar to helper cells, which mainly secrete cytokines such as IFN-γ, TNF-β and GM-CSF [23]. Researchers even further subgroup these cells into the NK1 and NK2 categories, consistent with Th1 and Th2, mainly secreting IFN-γ and IL-5, respectively [82].

Besides established cytotoxic cNK cells, it has been demonstrated that NK cells could differentiate into antigen-presenting NK (AP-NK) cells [83], helper NK (NKh) cells [84] and regulatory NK (NKreg) cells, each defined by surface molecules and individual functions. A new CD8αα^+^MHC-II^+^ phenotype with professional APC capacity was considered to represent unusual AP-NK cells, recognizing and eliminating autoreactive T cells and finally killing them like cNK cells [85]. Human plasmacytoid dendritic cells (DCs) activated by the preventive vaccine FSME upregulate CD56 expression on their surface [86]; in mice, B220^+^CD11c^int^NK1.1^+^ cells have antigen-presenting capacity like DC, hence their name interferon-producing killer DC [87, 88].

Invariant natural killer T cells (iNKT) constitute a subgroup of T cells expressing NK cell-markers. Activated by CD1d-presenting antigens, NKT could secrete not only Th1-type but also Th2-type cytokines to participate in immunity [89, 90]. Th1-polarized iNKT cells exhibit a tumor-depletion phenotype, and Th2-polarized iNKT cells contribute to tumor progression, similar to polarized T cells [91, 92]. Recent studies also highlighted new functional subtypes of iNKT cells. However, in recent years, due to their close relationship with innate immunity, iNKT cells are potentially defined as a special subgroup of ILCs.

### NK cells in the tumor microenvironment

#### Conventional roles of NK cells in immunity

**Fig. 2:**
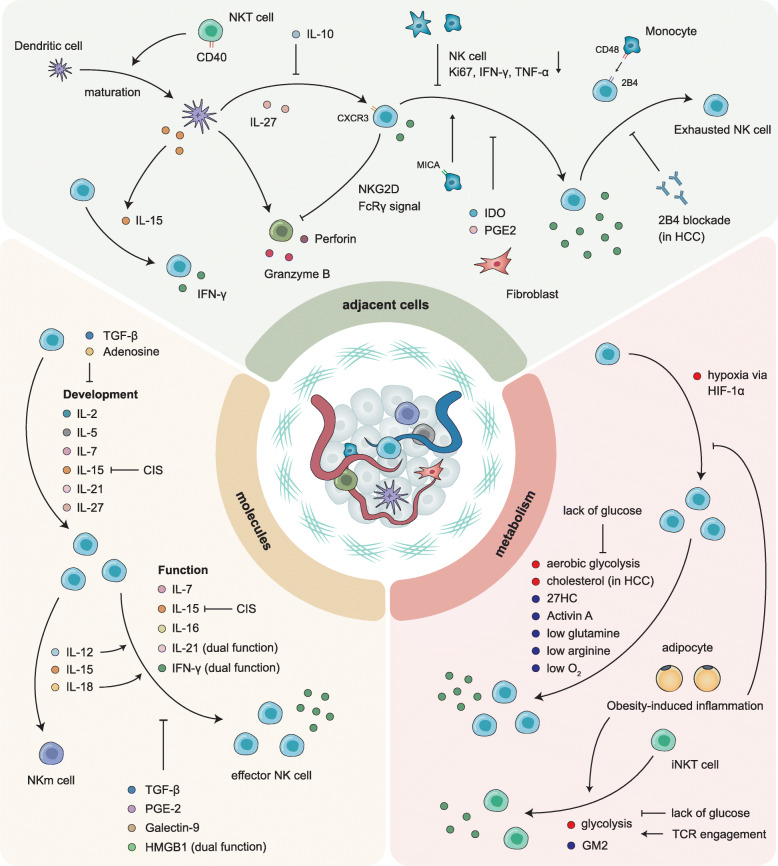
The complex interaction between NK cells and the extracellular matrix. Exposure of NK cells to the adjacent cells, molecules and metabolites in the extracellular matrix affects their development, maturation, activation and functions. CXCR3, C-X-C motif chemokine receptor 3; NKG2D, nature-killer group 2, member D; IFN-γ, interferon γ; TNF-α, tumor necrosis factor α; IDO, indoleamine 2,3-dioxygenase; MICA, MHC class I polypeptide-related sequence A; PGE2, prostaglandin E2; HCC, hepatocellular carcinoma; CIS, cytokine-inducible SH2-containg protein; TGF-β, transforming growth factor-β; HMGB1, high-mobility group box 1; HIF-1α, hypoxia inducible factor-1α; 27HC, 27-hydroxycholesterol; iNKT, invariant natural killer T; GM2, β-N-acetylhexosaminidase; TCR, T cell receptor

Detection of aberrant cells by NK cells is determined by the intergradation of complex signals such as IL-12, IL-15 and IL-18 [93, 94], and the balance between activating and inhibitory signals interacting with MHC-I on the surface of target cells (Fig. 2). During infection and inflammation, NK cells are recruited and activated in a short period of time, proliferate quickly and contribute largely to both innate and adaptive immune responses [8, 95]. Except for their newly proven regulatory effects, NK cells were first found to directly target infected cells or foreign pathogens; therefore, deficiency in NK cells in both mice and humans results in susceptibility to many viral infections and adverse clinical outcomes, validated by clinicians and researchers.

Similar to other innate immune cells that are unable to accurately recognize target cells, NK cells rely on other stromal cells, including DC, which trans-presents IL-15 for NK cell activation [96], and MICA-expressing monocytes, which bind to Fc receptor to enhance antitumor function [97], to fully differentiate and induce effector responses, but surprisingly possess the ability to form immunological memory, termed “trained immunity”. Once considered as a hallmark of adaptive immunity, in recent years, the phenomenon of immunological memory has also been found in innate immune cells, especially in the myeloid lineage, e.g., monocytes and macrophages. In addition, mounting evidence indicates that in humans, NK cells can remember previous exposure to inflammatory microenvironment, and occurrence of similar cytokines could induce trans-differentiation from normal NK cells to memory NK (NKm) cells [98–101]. This was evidently observed in response to viral infection in humans, prompting the development of NK cell-based vaccines to generate potent effects toward diseases [102, 103]. A large number of NKm cells are observed in the tumor microenvironment, producing high levels of IFN-γ, perforin and granzyme family molecules after re-stimulation [104]. However, concerning to tumors, dysfunction of NK and NKm cells is emerging as an indispensable and undeniable event, leading to not only proliferation of tumor cells but also the formation of distant metastases [101]. It was observed that repeated exposure of NK cells to NK receptor ligand-expressing tumor cells (e.g. NKG2D) finally results in NK cell dysfunction, and effector responses cannot be stimulated in vivo [105, 106]. These results indicate that the formation of NKm cells may not just depend on target cell recognition through surface receptors, and certain cytokines (including IL-12, IL-15 and IL-18) could be key to this process.

Though the half-life of normal NK cells is only for 1–2 weeks, NKm cells can live for 3–4 weeks [107]. This long-term effect truly helps researchers better modulate the function of NK cells in protecting against tumors, and emerging results suggest that NK cells not only rely on MHC-I recognition but also depend on many other signals, shedding light on the use of NK cells and related signaling pathways as future treatment options.

#### Infiltration of NK cells with cancer genotypes and phenotypes

In 2000, an 11-year follow-up study of the Japanese general population, with rigorous use of various related biochemical and immunological markers, indicated that elevated cytotoxic activity of peripheral NK cells is positively associated with reduced cancer risk and vice versa, suggesting the certain importance of natural immune response toward tumors [108]. However, the specific role of NK cells remains controversial and largely depends on distinct cancer types [109]. Even in the same type of cancer, NK cells are highly heterogeneous, characterized by the abundance of surface receptors and the complexity of tumor intrinsic signaling pathways [95, 110]. In the CIBERSORT analysis, NK cells were thoroughly divided into resting and activated subtypes, each contributing to the formation of the tumor microenvironment [111]. In preclinical studies, NK cells were shown to indicate survival and thus therapeutic response in different types of cancer, as detected by immunohistochemistry, immunofluorescence or flow cytometry using different surface and functional markers (Table 2) [46, 56, 112–129]. Although CD3^−^CD16^+^CD56^+^ cells reflect different clinical outcomes in different cancers, functional molecule-positive NK cells, including NKp30^+^ and NKp46^+^, indicate favorable survival, pointing out the fact that full activation but not only infiltration density finally determines NK cell-associated immune response.

**Table 2:** Infiltration of NK cells in different cancer types and its influence on clinical outcome

Cancer type	Sample	Detection method	Marker	Clinical outcome	Ref.
Pancreatic cancer	Blood	Flow cytometry	CD3^−^CD16^+^CD56^+^	Adverse OS	[112]
Colorectal cancer	Tumor	IHC	CD57^+^	Favorable OF and DFS	[113]
	Lymph node	IHC	CD56^+^	Favorable RFS	[114]
	Blood	Flow cytometry	CD3^−^CD16^+^CD56^+^	Favorable OS	[115]
Chronic myeloid leukemia	Blood	Flow cytometry	CD3^−^CD16^+^CD56^dim^	Favorable molecular RFS after imatinib discontinuation	[116]
Chronic lymphocytic leukemia	Blood	Flow cytometry	CD3^−^CD16^+^ and/or CD56^+^	Favorable OS	[117]
Follicular lymphoma	Blood	Flow cytometry	CD3^−^CD56^+^ and/or CD16^+^	Favorable OS	[118]
Mantle cell lymphoma	Blood	Flow cytometry	CD3^−^CD16^+^ and/or CD56^+^	Adverse OS and PFS	[119]
Liver cancer	Tumor	IF	CD56^+^PD1^+^	Adverse survival	[120]
	Tumor	IHC	NKG2A^+^	Adverse OS and DFS	[46]
	Tumor	Flow cytometry	CD3^−^CD56^+^CD49a^+^	Adverse OS and DFS	[121]
	Tumor	Flow cytometry	CD3^−^CD56^+^CD96^+^	Adverse DFS	[56]
Prostate cancer	Blood	Flow cytometry	CD3^−^CD56^+^ NKp30^+^ or NKp46^+^	Favorable OS	[122]
Lung cancer	Blood	Flow cytometry	CD56^dim^CD16^+^NKp46^+^	Favorable OS	[123]
	Blood	qRT-PCR	NKp30	Adverse OS and PFS	[124]
	Tumor	IF	CD56^+^ and/or CD16^+^	Favorable OS	[125]
Breast cancer	Tumor	IHC	CD3^−^CD56^+^	Favorable DFS	[126]
	Tumor	IHC	CD56^+^	Adverse OS	[127]
Gastric cancer	Tumor	IHC	NKG2D^+^	Favorable OS	[128]
Bladder cancer	Tumor	Flow cytometry	CD45^+^CD14^−^CD19^−^CD3^−^ILT3^−^cKIT^−^CD56^bright^	Favorable OS and CSS	[129]

*Abbreviations: IHC* immunohistochemistry, *IF* immunofluorescence, *qRT-PCR* quantitative real time polymerase chain reaction, *OS* overall survival, *RFS* recurrence-free survival, *PFS* progression-free survival, *DFS* disease-free survival, *CSS* cancer-specific survival

In BC, besides the ability of total NK cells to reflect favorable survival, peritumoral abundance of NK cells also correlates with elevated pCR rate of neoadjuvant chemotherapy in large and locally advanced breast cancer [130], and vice versa. To address this, it is currently well accepted that tumors are highly heterogeneous, and even one histologic type can be separated into several molecular subtypes. BC can be thoroughly clustered into luminal, HER2-enriched and triple-negative types according to the expression of surface molecules, and both infiltration and activation status of NK cells vary by cluster, e.g., obviously elevated NKG2D in luminal tumors [131]. However, due to the complex and variable functional status of NK cells, their actual role in the TME still awaits further elucidation.

#### Underlying mechanisms of immune escape and metastasis of cancer

As noted above, NK cells rely on the balance between activating and inhibitory receptors to exert their killing effects, and perforin and the granzyme family of proteins are the main effector molecules. Consistent with cNK cells, as a newly-found subgroup of NK cells, activated NKT cells directly detect and kill CD1d^+^ tumor cells in several types of cancer [132, 133]. In addition, by expressing high levels of CD40, NKT cells induce DC maturation, thus activating CTL and cNK cells to enhance their anti-tumor effects [134, 135].

**Fig. 3:**
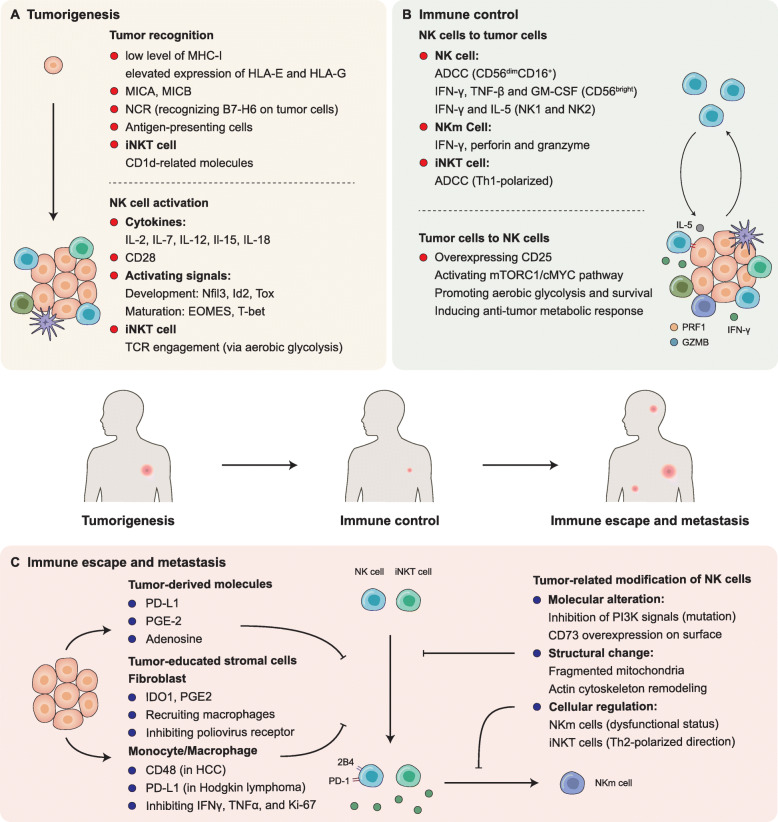
Interplay between cancer cells and NK cells during tumorigenesis. The interaction between tumor cells and NK cells changes continuously with NK cell development, tumor progression and metastasis. During the stage of tumorigenesis (**a**), NK cells recognize tumor cells through various surface molecules and switch to the active status. In the immune control stage (**b**), NK cells exert killing effects by ADCC, secreting cytokines and generating memory NK cells. Meanwhile, changes in the surface molecules of tumor cells also promote anti-tumor metabolic responses. However, long-term exposure of NK cells to tumor cells, tumor- derived molecules and tumor-educated stromal cells, including fibroblast, monocyte and macrophage, causes NK cells to be in an immunosuppressive state, thereby promoting tumor immune escape and metastasis (**c**). MHC- I, major histocompatibility complex-I; MICA, MHC class I polypeptide-related sequence A; MICB, MHC class I polypeptide-related sequence B; NCR, natural cytotoxicity receptor; Nfil3, nuclear factor interleukin-3-regulated protein; Id2, inhibitor of DNA binding 2; Tox, thymocyte selection associated high mobility group box; EOMES, eomesodermin; T-bet, T-box transcription factor 21; ADCC, antibody-dependent cell-mediated cytotoxicity; GM-CSF, granulocyte-macrophage colony stimulating factor; PRF1, perforin 1; GZMB, granzyme B; PD-L1, programmed cell death ligand 1; PGE-2, prostaglandin E2; HCC, Hepatocellular Carcinoma; IFN, interfron; TNFα, Tumor Necrosis Factor α;PI3K, Class IA phosphatidylinositol 3 kinase

Apart from primary tumor proliferation, metastasis remains lethal, accounting for the majority of cancer-associated deaths, and invasion-metastasis cascade is largely attributable to the immune escape [136]. With such rapid and effective ability to target tumor cells directly and indirectly, NK cells are suppressed by tumor-derived molecules, tumor-educated stromal cells (Fig. 2) and tumor cells (Fig. 3), eventually contributing to the progression and multi-step metastatic process of cancer. For example, single-cell analyses found that in lung adenocarcinoma, CD16^+^ NK cells are hardly infiltrated and present lower granzyme B and CD57 expression compared with normal lung tissue, appearing to form NK cell-excluded TME [137].

Class IA phosphatidylinositol 3 kinases (PI3Ks) are involved in growth and survival of normal cells, and mutation of the PI3KCA isoform is commonly found in the genomic landscape of many cancers. Inhibiting abnormally activating signals of PI3KCB with a tested inhibitor called P110β in hematologic malignancies obviously enhances susceptibility of tumors to NK cell activity in vitro, probably through the regulation of MHC-I [138]. Tumor-derived prostaglandin E2 [139], extracellular adenosine [140], fragmented mitochondria in the cytoplasm of tumor-infiltrating NK cells [141] and actin cytoskeleton remodeling [142] also lead to immunosuppression and help potentially metastatic cancer cells to avoid NK cell elimination. Interestingly, in a breast cancer cell line, CDC42 or WASP knockdown does not change the activation status of NK cells but obviously increases the expression of effective granzyme B and overcomes resistance to NK cell-mediated attack [142]. Further investigation in this field might help identify a new signaling pathway or a new marker of NK cell-activation and provide new insights into NK cell-therapy. Similar to the inhibitory role of CD73 in T cell-immunity, tumors can train normal NK cells into CD73^+^ NK cells which express high levels of checkpoint molecules, including LAG-3, PD-1, and PD-L1, finally resulting in immune escape [57]. As mentioned above, Th2-polarized iNKT cells in the TME contribute to tumor progression through immunosuppressive effects [91], and continuous exposure to ligands expressed on the surface of tumor cells induces the dysfunction of NKm cells that are engaged in long-term anti-tumor immunity [106]. Moreover, absence of NKG2D is a common feature of functionally suppressed NK cells, which is achieved through many different pathways [105, 143], thus could be used as a marker to guide NK cell-related therapies [144].

In addition, NK cell-associated cytotoxicity can also be impaired by stromal cells, for example cancer-associated fibroblasts, monocytes, macrophages and other immune cells. Fibroblasts in TME suppress function of NK cells through downregulating ligands of NK cell activating receptors [145], enhancing tumor-associated macrophages enrichment [146], and producing extracelluar matrix components such as IDO and PGE2 [147]. In human gastric cancer, tumor infiltrating monoctyes/macrophages can reduce IFNγ, TNFα, and Ki-67 expression of NK cells via TGFβ1, thus impairing NK cell function [148]. Meanwhile, the interaction of PD-1^+^ NK cells and PD-L1^+^ monoctyes/macrophages in Hodgkin lymphoma results in immune evasion, which can be reversed by PD-1 blockade [149]. The monocytes from hepatocellular carcinoma express CD48, which could block 2B4 on NK cells and induce NK cell dysfunction [150].

Consistent with CD8^+^ T cells, activated PD-1^+^ NK cells are inhibited by elevated PD-L1 expression in the TME [58]. Dysfunction of NK cell following surgery has been regarded as a risk factor of metastasis and can be partly explained by disturbing the balance between activating and inhibitory signals. NK cell-mediated metastasis control was found dependent on dectin-1-mediated activation of macrophages, especially the plasma membrane tetraspan molecule MS4A4A [151]. In colorectal cancer, lipid accumulation is common for postoperative patients and facilitates the formation of metastasis by impairing NK cell function, by elevating CD36 expression [152]. Hence, NK cells can be rarely seen in metastatic melanoma and are mainly TIGIT^−^CD226^−^ which are deprived of cytotoxicity toward MHC-I-defient malignant cells [153]. Interestingly, proved by convincing genetically engineered models, it is the absence of NK cells but not CD8^+^ T cells that evidently leads to the metastatic dissemination of small cell lung cancer, pushing us to better define the pivotal role of NK cells in both initial progression and later metastasis of cancer [154].

#### Relationship between NK cell-based and CD8^+^ T cell-based immunity

NK cells, though belonging to innate immunity, have characteristics similar to cytotoxic CD8^+^ T cells [95]. Originally proven important in the two-signal activation model of T cells, CD28 is also necessary for optimal cytokine secretion and proliferation of NK cells both in vitro and in vivo [155]. It was shown that IL-21 is important for the maturation and activation of NK cells [156–158]. However, data also uncovered the double-sided function of IL-21 in the development of NK cells and surprisingly, its positive role in T-cell-based immune response [159, 160], for example inducing KLRG1^+^CD8^+^ T cells during acute intracellular infection [161]. Besides IL-21, NK cells produce multiple other cytokines during their proliferation, maturation and function similar to T cells, including IL-2 [162], IL-7 [163] and IL-15 [164], validating the close relationship between these two types of effector cells in the body’s immune system.

Although sharing many similarities, compared with effector T cells, NK cells are more cytotoxic to tumors and possess lower immunogenicity [10]. As mentioned above, NK cells respond to target cells more quickly and do not need extra ligation of activating receptors [95]. On the contrary, NK cells have the ability of suppressing the function of CD8^+^ T cells via NKG2D in severe aplastic anemia [165]. It has been underlined that during infection with chronic lymphocytic choriomeningitis virus, NK cell-intrinsic FcRγ signaling could inhibit the expansion of CD8^+^ T cells [166]. Interestingly, tumor cells that develop checkpoint blockade resistance to CTLs, especially through suppressed MHC-I expression, are more vulnerable to NK cell-based immunity; thus, combination immunotherapy utilizing both NK cells and CD8^+^ T cells can constitute a future strategy in terms of tumor immune escape [167]. Besides, in tumors that lack MHC-I-related molecules, elevated amounts of HLA-E and HLA-G were observed, indicating the possibility for NK cells to harness this unclassical pathway [168]. More basic researches are urgently needed for better understanding of the complex relationship between these two major effector cells, as NK cells-based treatment is currently highly underestimated.

### Crosstalk of NK cells and metabolic signaling in cancer

#### Immunometabolic disorder as a hallmark of cancer

Similar to high blood pressure and diabetes, cancer is currently regarded as not only a process of pathogenesis but also a social issue. Lipid accumulation in the liver, abnormal glucose metabolism and irregular lifestyle all contribute to tumorigenesis and cancer progression, which eventually prompt research about the underlying mechanisms of this phenomenon. It is admitted that the metabolic competition between tumor and stromal cells largely affects the process of tumorigenesis and cancer progression. In the TME, NK cell function is impaired not only by suppressive cytokines but is also attributable to inappropriate metabolic conditions, including hypoxia, lack of nutrition and abnormal concentrations of tumor-derived products such as lactate, which induces unfavorable acidic condition, hindering the proliferation and cytokine production of CTLs as well (Fig. 2) [169]. As metabolic disorder is currently considered a hallmark of cancer, which shares a close relationship with the microenvironment, the idea of harnessing immunometabolism attracts increasing attention to improve the efficacy of NK cell-dependent anti-tumor therapy.

The normal breast tissue is surrounded by adipose tissue, and obesity is considered a potential risk factor for breast cancer, which is supported by population-based studies, together with high mental pressure, evidently affecting lipid and glucose metabolic pathways. Obesity-induced inflammation in adipose tissue could result in the recruitment of M1-polarized macrophages, neutrophils, NK cells and CD8^+^ T cells, and higher expression levels of pro-inflammatory cytokines, as well as obvious exclusion of Treg and invariant NKT (iNKT) cells [170]. In 2011, aware of the importance of tumor-promoting inflammation in the TME, Weinberg et al. included this phenomenon into the hallmarks of cancer and particularly highlighted inflammation induced by the innate immune response [1]. Thus, improved understanding of the mechanism by which metabolic activity affects the function of tumor-infiltrating stromal cells, finally resulting in cancer progression and immune escape, would provide clues for developing novel therapeutics for immunometabolic targets.

#### Metabolic disorder of conventional NK cells in the TME

NK cell function can be altered by different components in the TME. Breast cancer metabolomics data overtly show that lipid and glucose metabolic pathways are highly activated, especially the fatty acid synthase glycolysis pathway, compared with paired peritumoral tissue.

Similar to other lymphocytes, NK cells require energy to survive, and glucose consumption is evidently increased after full activation, while the competition between NK cells and tumor cells could disturb such need. It has been shown that surface transporters, especially glucotransporter 1 (GLUT1), help NK cells utilize glucose to generate ATP and pyruvate, contributing to glycolysis and oxidative phosphorylation [171, 172]. Several studies have pointed out the importance of sufficient glucose supply for NK cell activities, including proliferative capacity, activation status, cytokine production and direct cytotoxicity [173, 174]. Glycolysis and oxidative phosphorylation contribute to maintain the cytotoxic ability of NK cells, as their inhibition highly decreases the expression levels of IFN-γ and Fas ligand [175]. NKG2D is essential for the activation of NK cells, which relies on glycolysis. Researchers have identified several pathways pertaining to the interlinked metabolic activity and NK cell function. Obesity-related inflammation is dependent on the IL-6/Stat-dependent pathway, thereby resulting in a distinct functional status of NK cells [176]. In addition, Assmann et al. highlighted that sterol regulatory element-binding proteins (SREBP) transcription factor-controlled glucose metabolism is essential for metabolic reprogramming in activated NK cells, providing new insights into this process [177]. Accordingly, SREBP inhibitors such as 27-hydroxycholesterol (27HC) are accumulated in the TME, partly affecting SREBP-related glycolysis in ER-positive BC [178]. However, as most studies only focus on GLUT1, other transporters and unclassical pathways should also be paid attention to, paving the way for deeper understanding of the complex relationship between glucose metabolism and NK cell function.

Hypercholesterolemia remains a risk factor for ER-positive BC. In 2013, 27HC, which negatively regulates SREBP, was also found by Nelson et al. to be a bridge linking hypercholesterolemia and BC [179]. It was also found that treating mice submitted to high-cholesterol feed with an inhibitor of CYP27A1, an enzyme important in 27HC biosynthesis, obviously decreases the number of metastases in mice, which reverses immune suppressive environment [180]. Therefore, using drugs designed to decrease blood cholesterol or directly inhibiting the formation of 27HC could be a potential strategy for patients with ER-positive BC. Surprisingly, a recent study suggested that high serum cholesterol and cholesterol accumulation in NK cells increase their anti-tumor ability by facilitating the formation of lipid rafts in the liver-tumor-bearing murine model [181], highlighting the heterogeneous functions of lipid metabolism in cancer.

Hypoxia is also a common feature of cancer, and often mentioned concurrently with low pH in the TME. Previous findings indicated that besides promoting tumorigenesis and cancer progression, hypoxia also stimulates NK cell formation via HIF-1α, initiating a conflict between suppressing and activating this signal [182]. Studies have also shown that low O_2_ in TME harms the function of NK cells by downregulating activating signals such as NKG2D, NKp30 and CD16, thereby limiting cytokine production and cytotoxicity and resulting in metastasis [183, 184]. In addition to regulating intracellular signals directly, the hypoxic microenvironment could degrade NK cell-secreted functional molecules such as granzyme B [185], together with CTL-based immunity, which is partly rescued by IL-2 [186, 187]. Considering the pivotal roles of interleukin family members in the maintenance of NK cells, we paid more attention to these cytokine-primed metabolic pathways and found more clues under hypoxic conditions.

Besides, tumor cells can directly alter the metabolic status of NK cells. This can be positive as CD25 expression on NK cells is overexpressed after interaction with tumor cells, inducing long-term anti-tumor metabolic responses by promoting glycolysis and NK cell survival, supported by mTORC1/cMYC signaling activation. However, worsening occurs later as the harmful effects overcome the positive impact. For instance, glutamine addiction and high consumption of nutrients remain common in BC. In vitro studies showed arginine deficiency inhibits IFN-γ production by primary human NK cells [188]. Additionally, mTOR signaling within NK cells can be largely suppressed under low-arginine or glutamine conditions, which also affect IL-2-related stimulation process via cMYC [189]. Upon direct contact with tumor cells to form an immune synapse in response to local energy consumption, mitochondria of NK cells are depolarized and lose metabolic energy [190]. Inhibiting PPARα/δ or blocking the transport of lipids into mitochondria reverses NK cell metabolic incapability and restores cytotoxicity [191].

Indeed, previous studies have reported that anti-PD-L1 therapy could reshape metabolic pathways in the tumor microenvironment and re-stimulate exhausted CD8^+^ T cells for cytotoxicity [192]. Interestingly, NK cells also express the ligands of these checkpoints. Glycoengineering of NK cells enhances their killing ability toward CD22^+^ lymphoma in a CD22-dependent manner [193]. Blockade of monocarboxylate transporter 1, which regulates cell metabolism, using AZD3965 also potentiates NK cell activity [194]. Studies that focus on translating mature theories into the practical use of NK cells are promising.

#### Aberrant metabolic features of iNKT cells

As mentioned above, iNKT cells can be polarized into different properties, each possessing distinct functions. The normal breast tissue is surrounded by adipose tissue. Different from conventional T cells, iNKT cells comprise large amounts of stromal cells in adipose tissue, whose infiltration decreases apparently in high-BMI individuals [195, 196].

With invariant TCR on the surface, iNKT cells are termed innate-like T lymphocytes and act on the front line of the immunity battle against cancer [197, 198]. iNKT cells recognize glycolipid signals but not peptides via semi-invariant TCR, and are restricted to glycolipid antigens presented via CD1d-related molecules, which are MHC-like and highly enriched, especially in adipocytes and hepatocytes, linking innate and adaptive immune responses. This process can be altered by metabolic activity. GM2 is a glycosphingolipid that binds the CD1d molecule. Pereira et al. pointed out that GM2 inhibits the activation of iNKT cells in a dose-dependent manner, which might result from its competition with α-GalCer for binding CD1d [199]. Compared with T lymphocytes, iNKT cells show much higher capacity of glycolysis but reduced mitochondrial respiratory activity, resulting in particular molecular features. Under hypoxia, widespread RNA editing can be induced by mitochondrial respiratory inhibition via APOBEC3G, an endogenous RNA editing enzyme [200]. Fu et al. demonstrated that aerobic glycolysis in iNKT cells is highly increased after TCR engagement, which is essential for the production of IFN-γ [90]. This process can also be inhibited by the lack of glucose. Hence, reduced expression of IFN-γ in iNKT cells compared with the normal tissue was confirmed in several tumor types, predicting patient response to the therapy of PD-1 blockade [201–203]. In humanized mouse models undergoing PD-1 blockade and CAR-T (with different costimulatory molecules) combination therapy, only those with Δ-CD28 CAR control tumor growth, and in vitro analysis showed that these cells exhibit elevated glycolysis, fatty acid oxidation and oxidative phosphorylation [204]. Overcoming the impaired metabolic function combined with immune checkpoint blockade would be a potential strategy in future researches and clinical practice.

However, what is currently known about iNKT cells is just the tip of the iceberg. Compared with T lymphocytes, it remains unclear how intracellular metabolic signals influence the survival and function of iNKT cells, which deserves further investigation, as this may be the next potential target of cancer immunometabolic therapy after CD8^+^T cells and NK cells.

### NK cells in cancer therapy

As an important effector of innate immunity, though suffering a resistance evolved by tumor cells, NK cells show their potential to be used in clinical practice [205–207]. In the past few years, researches about NK cell-related immunotherapy have flourished and the latest development mainly focused on cytokine supplement, monoclonal antibody, modification of internal signal pathway, adoptive transfer and genetic engineering of NK cells. Besides, NK cell-based therapy has achieved favorable results used either alone or in combination with other therapies, which suggests a wide and effective use in malignancies.

#### Cytokine supplement

**Fig. 4:**
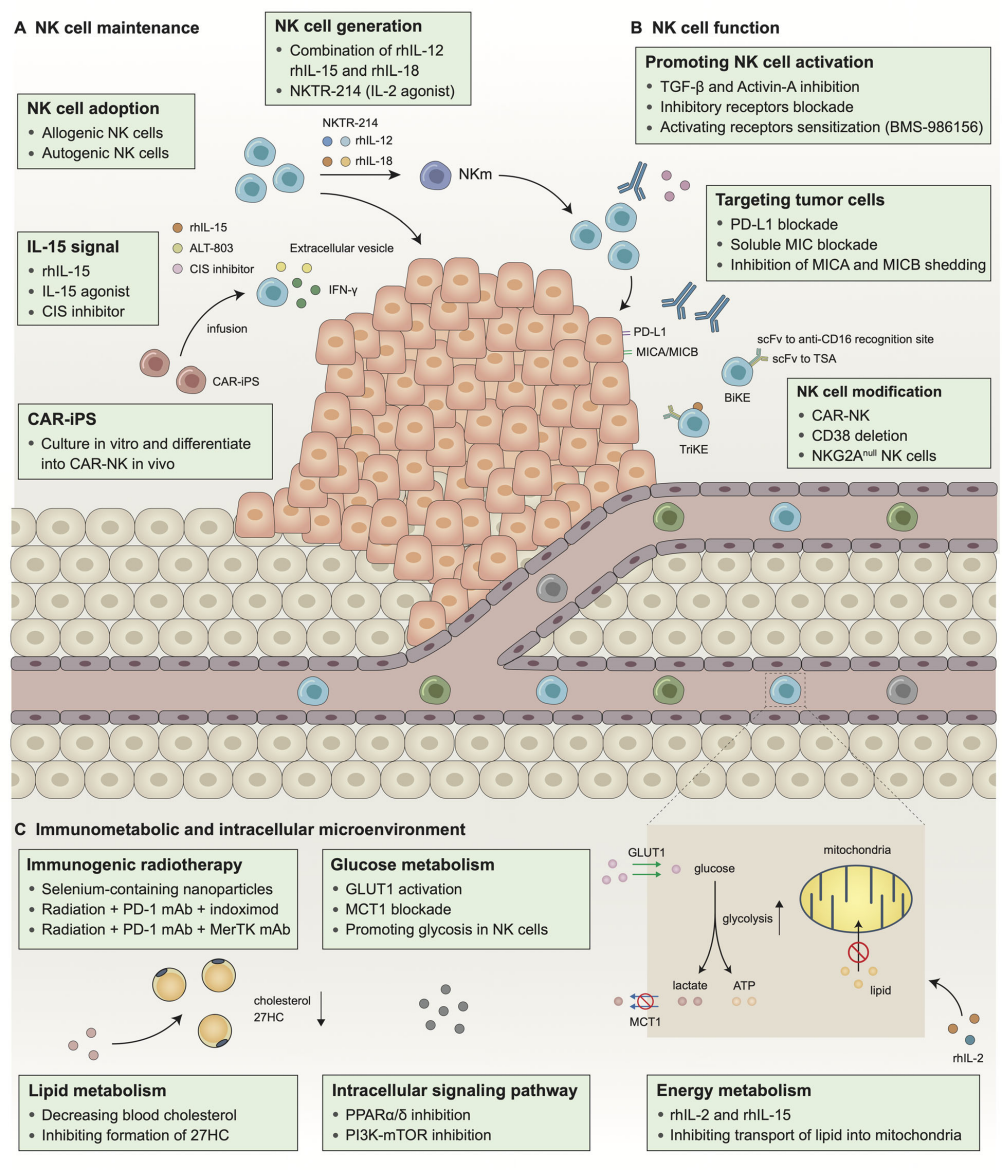
Possible targets harnessing NK cells in cancer therapy. In order to obtain better clinical efficacy and reduced severe adverse events, the development of NK cell-based therapies that support NK cell maintenance (**a**), enhance NK cell function (**b**) and harness abnormal immunometabolic and intracellular microenvironment (**c**) is essential. rhIL-12/15/18, recombinant human interleukin-12/15/18; CAR-iPS, chimeric antigen receptor-induced pluripotent stem cell; MIC: MHC I chain related molecule; MICA, MHC class I polypeptide-related sequence A; MICB, MHC class I polypeptide-related sequence B; PD-L1, programmed cell death-ligand 1;scFv, single-chain variable fragment; TSA, tumor specific antigen; BiKE, bispecific killer cell engager; TriKE, trispecific killer engager; CAR-NK, chimeric antigen receptor-nature kill; PD-1, programmed cell death protein 1; MerTK, MER proto-oncogene, tyrosine kinase; 27HC, 27-hydroxycholesterol; GLUT1, glucotransporter 1; MCT1, monocarboxylate transporter 1

IL-15 promotes the development and cytotoxic ability of NK cells, and several clinical trials have illustrated the safety profile of recombinant human IL-15 (rhIL-15) in multiple tumors [208, 209] as well as its agonist, ALT-803, in metastatic lung cancer and post-transplantation patients (Fig. 4) [210, 211]. In an open-label, phase Ib trial, ALT-803 showed a fantastic potential when combined with anti-PD-1 monoclonal antibody (nivolumab) without increasing the incidence of very severe grade 4 or 5 adverse events [211], which evidently could be the future way to enhance rhIL-15 treatment. In addition to soluble IL-15 in the microenvironment, it has been revealed that in the mouse, direct contact with membrane-bound IL-15 on adjacent stromal cells could induce stronger cytotoxic effects in NK cells [212]. Heterodimeric IL-15 can also increase intratumoral NK cell and CD8^+^ T cell infiltration, elevating the effective rate of current immunotherapy [213].

Apart from IL-15, other interleukins are also synergetic to this process (Table 3). IL-21 enhances tumor rejection in mice via NKG2D-dependent NK cell activity, suggesting IL-21 to be a possible target for immune escape induced by NKG2D elicitation [214]. However, IL-15-dependent expansion of resting NK cells can be suppressed by IL-21, while on the other hand adaptive immune response is enhanced [159], providing substantial insights into this complex network in clinical use. Besides, blocking CIS could promote IL-15-type cytotoxicity and thus results in increased production of IFN-γ [22]. NK cells pre-exposed to IL-12, IL-15 and IL-18 accumulate in the tumor tissue and retain their anti-tumor function both in vitro and in vivo. However, IL-15 alone does not exert such effects [104]. In addition to the inner pathway, treatment with IL-2 and IL-15 obviously enhances glycolysis and oxidative phosphorylation of NK cells, thus promoting the killing ability. In a first-in-human phase I multicenter study, NKTR-214, a novel IL-2 pathway agonist, was found promoting proliferation and activation of NK cells without expansion of Treg cells [215]. For metastatic melanoma refractory to CD8^+^ T cell cytotoxicity due to the lack of MHC-I, combination of IL-15 and TIGIT blockade shall be effective by stimulating NK cell-mediated immunity [153].

**Table 3:** Clinical trials for established NK cell-related therapies

Mechanism	Condition	Intervention	Phase	Trial identifiers
IL-15 signal pathway	Metastatic malignant melanoma, RCC	Recombinant human interleukin-15(rIL-15)	I (first-in human)	NCT01021059
	Advanced metastatic solid tumor	IL-15 by continuous infusion	I	NCT01572493
	Refractory and relapsed adult T cell leukemia	IL-15 + alemtuzumab (anti-CD52)	I	NCT02689453
	Refractory and relapsed chronic lymphocytic leukemia	IL-15+ obinutuzumab (anti-CD20)	I	NCT03759184
	Hematologic malignancies recurring after transplantation	ALT-803 (IL-15 superagonist)	I (first-in human)	NCT01885897
	Metastatic NSCLC	ALT-803 + Nivolumab (anti-PD-1 antibody)	Ib	NCT02523469
IL-21 signal pathway	Relapse/refractory low-grade B-cell LPD	Recombinant human interleukin-21 (rIL-21) + Rituximab (anti-CD20 antibody)	I	NCT00347971
	Metastatic malignant melanoma, RCC	rIL-21	I	NCT00095108
	Stage IV malignant melanoma without prior treatment	rIL-21	IIa	NCT00336986
IL-12 signal pathway	Metastatic solid tumors	NHS-muIL12 (two IL12 heterodimers fused to the NHS76 antibody)	I (first-in human)	NCT01417546
	Murine mammary/subcutaneous tumors	NHS-muIL12+ Avelumab (anti-PD-L1 antibody)	Preclinical models	–
IL-2 signal pathway	Locally advanced or metastatic solid tumors	NKTR-214 (IL-2 pathway agonist)	I/II	NCT02869295
	Advanced Solid Tumors (Japanese)	NKTR-214 + Nivolumab	I	NCT03745807
Anti-KIR antibody	AML in FCR	IPH2101 (anti-KIR antibody)	I	EUDRACT 2005–005298-31
	Relapsed/refractory MM	IPH2101	I	NCT00552396
	Smoldering MM	IPH2101	II	NCT01248455
	Relapsed/Refractory MM	IPH2101+ lenalidomide (immunomodulatory agent)	I	NCT01217203
	AML	Lirilumab (2nd generation anti-KIR antibody))	II	NCT01687387
	SCCHN	Lirilumab + Nivolumab	II	NCT03341936
	Cisplatin-ineligible muscle-invasive bladder cancer	Lirilumab + Nivolumab	Ib	NCT03532451
Anti-NKG2A antibody	Advanced gynecologic malignancies	Monalizumab (IPH2201, anti-NKG2A antibody)	I	CCGT-IND221
	metastatic microsatellite- stable colorectal cancer	Monalizumab + durvalumab	First-in human	NCT02671435
	recurrent or metastatic head and neck cancer	Monalizumab + cetuximab	I	NCT02643550
TNF pathway	Advanced solid tumors	BMS-986156 (glucocorticoid-induced TNF Receptor-Related Protein Agonist) +/− Nivolumab	I/IIa	NCT02598960
Cell adoptive therapy	Canine sarcomas	Radiotherapy+ intra-tumoral autologous NK transfer	first-in-dog	–
	Recurrent medulloblastoma and ependymoma (children)	ex-vivo-expanded NK cells	I	NCT02271711
	Metastatic gastrointestinal carcinoma	Adoptive transferred autologous NK cells + cetuximab	I	NCT02845999
	HER2-positive cancers	Adoptive transferred autologous NK cells + trastuzumab	I	NCT02030561
	Locally advanced colon carcinoma	Adoptive transferred autologous NK cells + chemotherapy	I	–
	Malignant lymphoma or advanced solid tumors.	Adoptive transferred allogeneic NK cells	I	NCT01212341
	Myeloid leukemia	Adoptively transferred memory-like NK cells induced by IL-12, IL-15, and IL-18	I (first-in human)	NCT01898793
	High-risk AML, MDS, CML	MbIL21 ex vivo-expanded donor-derived NK cells	I	–
	MDS, AML.	Fludarabine/cyclophosphamide + total lymphoid irradiation + adoptive transferred IL2-activated haploidentical NK cells	I	EUDRACT 2011–003181- 32
	Older AML patients	Transferred umbilical cord blood CD34 hematopoietic stem + progenitor-derived NK Cells	I (first-in human)	Dutch clinical trial registry (NTR 2818)
	Non-Hodgkin lymphoma	Haploidentical donor NK cells + rituximab+ IL-2	II	NCT01181258
	Myeloma	α-galactosylceramide-loaded monocyte-derived dendritic cells + low-dose lenalidomide (mediate antigen-specific co-stimulation of human iNKT cells)	I	NCT00698776
CAR-NK therapy	CD19-positive lymphoid tumors	NK cells expressing anti-CD19 CAR, IL-15 and inducible caspase 9	I/II	NCT03056339

*Abbreviation: RCC* renal cell cancer, *NSCLC* non-small cell lung cancer, *LPD* lymphoproliferative disorders, *AML* acute myeloid leukemia, *FCR* first complete remission, *MM* multiple myeloma, *SCCHN* squamous cell carcinoma of the head and neck, *MDS* myelodysplastic syndromes, *CML* chronic myeloid leukemia

As mentioned above, cytokines (especially IL-12, IL-15 and IL-18) are critical to the formation of NKm cells. Memory-like NK cells supplemented with IL-12, IL-15 and IL-18 also show enhanced responses against acute myeloid leukemia both in vitro and in vivo [107], and are currently assessed in a first-in-human clinical trial. However, studies also pointed out that IL-12 could increase NKG2A expression and inhibit the activation of NK cells [216].

#### Monoclonal antibodies

As shown previously, in parallel with CD8^+^ T cells, NK cells can also be suppressed by immune checkpoint molecules. After cetuximab treatment, PD-1^+^ NK cells are more enriched in the TME and are correlated with favorable clinical outcome in head and neck cancer patients, which was further demonstrated by in vivo experiments and a stage III/IVA clinical trial assessing neoadjuvant cetuximab (NCT01218048) [58]. With PD-1 blockade (nivolumab), cetuximab-induced NK cell activation and function are remarkably enhanced in PD-L1^high^ tumors.

In addition to already-well-defined PD-1 and PD-L1, NK cells with reduced amounts of T-cell immunoglobulins and ITIM domain (TIGIT) show higher levels of cytokine secretion, degranulation activity and cytotoxicity [217], and blockade of TIGIT could prevent exhaustion in NK cells [20]. A recent study also highlighted that TIGIT-depleted NK cells are highly sensitized [218] and develop resistance to MDSC-mediated immunosuppression [219]. Meanwhile, CD96, which shares the same ligand CD155 with CD226 and TIGIT, negatively controls the immune response by NK cells [220] and predicts adverse survival in human hepatocellular carcinoma [56]. Single use of CD96 antibody promotes NK-cell-induced anti-metastatic ability [221], and such effect is largely increased when combined with anti-CTLA-4, anti-PD-1 or doxorubicin chemotherapy [222].

Though prospective, unexpected biological events have been observed in a single-arm phase II study showing that intravenous infusion of 1 mg/kg IPH2101 (a human monoclonal antibody against KIRs) results in severe contraction and obvious inhibition of NK cells in myeloma patients [223]. Lirilumab, a 2nd generation antibody targeting KIR, had encouraging results in a phase I trial, which demonstrated its safety [224]; however, the subsequent phase II trial in AML patients showed no clinical effects. Combination of CIS inhibition with CTLA-4 and PD-1 blockade exerts even greater effects in reducing melanoma metastasis compared with either of these treatments administered alone; thus, CIS inhibition could offer an alternative therapeutic option for patients not responding to other immune checkpoint inhibitors [22].

As an essential receptor for the activation of NK cells, NKG2D is blocked by many ligands (e.g., MICA, MICB, and ULBP1–6) upregulated in tumor cells as a result of abnormal cellular stress in the TME. A recent study demonstrated that antibodies targeting MICA and MICB can prevent NK cell recognition and tumor cell binding, inhibiting tumor growth in fully immunocompetent mouse models as well as humanized mouse models [129]. Furthermore, combination treatment targeting soluble MIC, e.g., MICA and MICB, and PD-L1 shows better effect than monotherapy in vivo [225]. Moreover, soluble MULT1, a high affinity mouse NKG2D ligand stimulates NKG2D in distant NK cells and enhances NK cell tumor immunity [106]. Hence, a clinically used antibody, monalizumab, has been developed targeting NKG2A, an inhibitory checkpoint of NK cells, which not only promotes NK cell function in various preclinical models, as previously characrerized, but also potentiates anti-PD-1 [226] and anti-EGFR (cetuximab) therapy [227]. In addition to antibody, NKG2A^null^ NK cells, constructed through retroviral transduction of NKG2A blocker which inhibits de novo NKG2A expression, present increased anti-tumor activity in pre-clinical model [228].

In summary, besides the targets close to T cells, including FDA-approved anti-CTLA-4 (ipilimumab) and anti-PD-1 (nivolumab, pembrolizumab) antibodies, others designed specifically for NK cells are also under clinical trials, e.g., anti-KIR (IPH2101, lirilumab) and anti-NKG2A (monalizumab) (Table 3) (Fig. 4).

#### Cell adoptive therapy and newly arising genetic modification of NK cells

As an applicable option for enhancing autologous immunity, adoptive transfer of NK cells has been implemented to treat certain types of cancer in the past few years [229]. Previous studies of NK adoptive transfer in acute myeloid leukemia patients have presented slightly beneficial effects in controlling disease [230, 231], and a phase II clinical trial in patients with recurrent ovarian or breast cancer showed that adoptive transfer of haploidentical NK cells after lymphodepleting chemotherapy leads to a temporary benefit but its clinical value remains controversial, and is partly limited by recipient reconstitution of regulatory T cells [232]. Apart from adults, a phase I clinical trial applied autologous ex-vivo-expanded NK cells to children with recurrent medulloblastoma and ependymoma and obtained good safety and therapitic efficacy [233]. Interestingly, transfer of NK cells along with CD34^+^ hematopoietic stem cells shows no added adverse effects but potential therapy response in older patients with acute myeloid leukemia [234]. Combinational application of NK cell infusion with monoclonal antibody provides a new direction of combinational immunotherapy. In a phase I trial, activated autologous NK cell were infused into patients with HER2-positive solid tumor undergoing trastuzmab and showed prelimitary anti-tumor phenotype [235].

In 2009, Fujisaki et al. found that overexpression of telomerase reverse transcriptase could lead to over 100 additional doubling cycles in NK cells, revealing a potential way to overcome the limitation of NK cell amplification in vitro [236]. However, though the adoption of NK cells seems promising in preclinical and clinical researches, many questions exist, e.g., the undeniable fact that NK cells gain self-renewal ability following infusion. Originally designed and considered a one-time-use therapeutic process, a small part of NK cells surprisingly remain alive and proliferate in the human body for months, mediating continuous surveillance against tumor [237]. However, in addition to beating tumor cells, the risk of long-lived NK cells should not be ignored where their “brake” is lost and they might also kill normal cells, even worse, finally increasing the possibilities of NK lymphoma [95, 238]. Hence, the ability of transferred NK cells may be limited by inappropriate persistence or expansion in vitro, and biology-driven methods are commonly used to overcome this issue before adoptive transfer. A recent study indicated that NK cells pre-activated by IL-12, IL-15 and IL-18 suppress graft-versus-host disease but obviously inhibit the generation and function of CD8^+^ T cells, which could result from mutual competition of IL-2 [239], limiting its value in the field of cancer treatment. The use of mouse models to unveil the detailed features of adoptive NK cells following in vitro proliferation would actually provide deeper insights into this treatment strategy, shedding light on the future implementation of NK cell-based therapy toward cancer.

Genetic modification of immune cells by chimeric antigen receptors (CARs) to target tumor cells directly is a promising therapeutic option in cancer therapy. Kymriah (CTL019, a CAR-T product) by Novartis was approved by the FDA for treating recurrent and refractory acute lymphoblastic leukemia in 2017, and remains under investigation for other indications in several clinical studies [240–242]. Two months later, Yescarta by Kite Pharma was approved for diffuse large B cell lymphoma [243]. Due to serious adverse effects induced by CARs, especially cytokine releasing storm (CRS) and neurotoxicity, Actemra (tocilizumab, anti-IL-6 monoclonal antibody) was then approved for CRS, with a further study also showing potential application of Anakinra (an antagonist IL-1 receptor) in such case [244, 245]. In NK cells, antibody engineering approaches optimize NK cell-mediated ADCC to tumor cells through the bispecific killer cell engager (BiKEs) or trispecific killer engager (TriKEs) antibodies (Fig. 4). BiKE connects a single-chain variable fragment (scFv) to the anti-CD16 recognition site with the scFv of a tumor specific antigen, such as CD19/CD20 for non-Hodgkin lymphomas, CD33/CD123 for acute myelogenous leukemia/AML and CD30 for Hodgkin lymphoma, to enhance NK cell recognition of tumor cells [246]. TriKE consists of a BiKE and cytokine IL-15, which boost NK cell function and survival. It was shown that CD19-CD16 BiKE engineered NK-92 cells are sufficient to overcome NK cell resistance in B-cell malignancies [247]. Meanwhile, CD16-IL15-CD3 TriKE can activate suppressed NK cells and induce NK cell-mediated control of MDS and AML [248]. The advantages of CAR-NK therapy are obvious, including higher possibility of recognizing tumors (including cytokines and apoptosis) and lower incidence of CRS compared with CAR-T (Table 3) [249, 250]. In 2018, Enli Liu et al. transduced cord blood-derived NK cells with a retroviral vector incorporating the genes for CAR-CD19, inducible caspase-9-based suicide gene (iC9) and IL-15, and demonstrated the efficacy and safety in cell lines and the murine model. In the engineered NK cells, CAR-CD19 redirected the specificity of NK cells against leukemia, IL-15 promoted NK cell proliferation, and iC9 allowed NK cells to initiate suicide after killing the target cells [251]. Since CAR-NK cells exhibit striking efficacy and limited toxicity, clinical trials assessing these CAR-NK cells have been launched. In recent phase I and II trials, iC9/CAR-CD19/IL-15 NK cells were prepared ex vivo and infused into patients with relapsed or refractory CD19-positive cancers after lymphodepleting chemotherapy. Among the 11 treated patients, 8 had an objective response, including 7 with complete remission, without major toxic effects [250]. Apart from engaging NK cell with CAR, geneic modification involves deleting surface molecules, e.g., CD38, on NK cells, which evidently elimates fratricide and enhances cytotoxic ability [252].

However, limitations should be mentioned. Due to the complex process of producing CAR-NK cells, the current procedure is too expensive and the effects on solid tumors are far from being satisfactory. CAR-iPS might be a future direction, which can grow in vitro and differentiate into CAR-NK cells in vivo to directly enhance anti-tumor immunotherapy [253]. Recently, Zhu H et al. reprogramed NK cell metabolism by depleting *CISH* in human iPSC-derived NK cells and obtained satisfactory persistence and anti-tumor activity in vivo, which could be a novel method to generate CAR-NK from CAR-iPS [254].

#### Refinement of the established therapies

As an important immunosuppressive cytokine that promotes tumor progression, TGF-β and its pathway represent potential opportunities for anti-tumor drug development. The clinical modulation of TGF-β, which is achieved through small-molecule inhibitors and antibodies, is being investigated in a number of clinical trials [255]. As the safety and efficacy of TGF-β blockade therapy have been demonstrated, two studies independently showed that combination treatments of TGF-β blockade with anti-PD-1/PD-L1 therapies have synergistic effects on murine EMT6 breast mammary carcinoma and colorectal cancer [256]. It is admitted that TGF-β inhibits NK cell metabolism, proliferation, cytokine production, cytotoxicity and anti-metastatic functions through mTOR signaling in multiple cancers [257]. Activin-A, a member of the TGF-β superfamily, increases ILC1-like tissue residency features, reduces cytokine production, and suppresses proliferative and metabolic functions in both human and murine NK cells through an alternative SMAD2/3-related pathway with effects similar to those of conventional TGF-β pathway [258]. Therefore, targeting TGF-β, TGF-β superfamily, and their downstream pathways in NK cells may be promising treatment options that enhance the efficacy of current immunotherapies (Fig. 4). Apart from blockage of immunosuppressive cytokine, immunostimulatory agonist have been tested in clinical trials. In a recent phase I/IIa study, BMS-986156, a human glucocorticoid-induced TNF receptor-related protein agonist, appears to increase NK cell proliferation no matter applied with or without nivolumab, and has an adorable safety and efficacy profile [259].

Rapamycin, an inhibitor of the mTOR pathway, and its derivative everolimus are effective for treating breast cancer in clinical trials, including BOLERO-6 and PrE0102 studies, either alone or in combination with endocrine and chemotherapy [260, 261]. However, NK cells highly rely on PI3K-mTOR signaling pathway-dependent metabolic reprogramming to exert their anti-tumor effects. Therefore, in patients with both PI3K-mTOR pathway activation and NK cell-based microenvironment, such inhibitors should be cautiously employed.

In HER2-enriched breast cancer, besides HER2-targeted therapies, anti-GD2 also appears to be promising in preclinical studies. However, GD2-related pathways are essential for ADCC [262], also reducing the effectiveness of NK cells, which provides insights into the complex relationships among such networks to minimize off-target effects exerted by established therapies on immunotherapy.

Radiotherapy was found to kill cancer cells via inducing DNA damage, but recent studies found its ability to cause immunogenic cell death, named immunogenic radiotherapy [263]. There is a growing interest to combine immunogenic radiotherapy and immunotherapy, plus chemotherapy or target therapy. A triple-combination therapy which inhibits PD-1 and MER proto-oncogene tyrosine kinase plus radiotherapy, increases NK cells infiltration in abscopal TME [264]. Adding indoximod, an inhibitor of IDO pathway, to radiotherapy and PD-1 blockade also enhances NK cell activity and shows great clinical response [265]. Another representative drug of triple-combination is selenium-containing nanoparticles,which delivers doxorubicin to the tumor site and releases high energy rays. The rays not only kill tumor cells, promote doxorubicin release, but also produce seleninic acid which enhances NK cell function [266].

## Conclusions and perspectives

In this review, we draw a picture about the development and function of NK cells and emphasize their variant roles in cancer biology. NK cells performed anti-tumor immunity through their interplay with cancer cells, stromal cells and extracellular matrix, especially the metabolites. As tumor metabolism and tumor imunnity are both recent attractive research areas, we summarized relationship between NK cell and metabolism, which may provide ideas for cross study of these areas. NK cells often suffer resistance in TME and possible mechanisms have been illustrated in many researches, thus developing NK cell-based therapeutic strategies. In addition, clinical trials taking advantage of NK cells, either used alone or in combination with other therapies, have achieved promising results, paving the way for the future basic and clinical researches of the previously ignored but now prosperous NK cell-based cancer therapy and lighting up hope for patients resistant to current T cell-based immunotherapy.

Due to the rapid progress in understanding the TME, new concepts of immunotherapy keep emerging, which obviously helps promote the utilization of immune response for the treatment of cancer, especially the long forgotten innate component. Although previously considered to be characterized clearly, recent evidence shows that the accurate processes of differentiation, activation and generation of memory NK cells remain controversial. Most clinical trials related to NK cell immunotherapy are still in phases I and II, and mainly treat hematological malignancies. Although the clinically-proven safety of these drugs is helpful for the clinical transformation of NK treatment methods for solid tumors, which have broader prospect and wider application in the future, difficulties to overcome still exist. A future challenge for the implementation of NK cell-based therapy is to better define specific NK cell populations and to identify respective markers, as well as functional and regulatory pathways in each subgroup, thereby using different therapeutic strategies for the treatment of tumors infiltrated with different NK cells. Besides, to avoid off-target effects exerted by established anti-tumor drugs on the microenvironment to inhibit treatment effectiveness, more carefully selected combination therapies should be implemented in future clinical trials.

As most studies now take advantage of NK cells originating from blood, trNK cells are worth further investigating for implication in adoptive cell therapy especially for solid tumors. In the era of precision medicine, defining these questions could prompt new approaches that would permit selective regulation of anti-tumor versus pro-tumor response of NK cells. It is an exciting moment in which attention should be paid to this long-ignored cell population, and future possible targets for improved treatment options may harness tumor intrinsic pathways involving both extrinsic innate and adaptive immunity-related microenvironment.

## Data Availability

Not applicable.

## Abbreviations

27HC: 27-hydroxycholesterol
ADCC: Antibody-dependent cell cytotoxicity
AML: Acute myeloid leukemia
AP-NK: Antigen-presenting natural killer
BC: Breast cancer
BiKE: Bispecific killer cell engager
CAR: Chimeric antigen receptor
CD: Cluster of differentiation
CIS: Cytokine-inducible SH2-containg protein
cNK cell: Conventional natural killer cell
CTLA-4: Cytotoxic T-lymphocyte antigen 4
CTLs: Cytotoxic T cells
CRS: Cytokine releasing storm
DC: Dendritic cell
EOMES: Eomesodermin
ER: Estrogen receptor
FDA: Food and drug administration
FSME: Influenza, pneumococcal, herpes zoster, early summer meningoencephalitis
GAB3: GRB2-associated binding protein 3
GLUT1: Glucotransporter 1
GM2: β-N-acetylhexosaminidase
GM-CFS: Granulocyte-macrophage colony stimulating factor
HER2: Human epidermal growth factor receptor 2
HIF-1α: Hypoxia inducible factor-1α
HLA: Human leukocyte antigen
Id2: Inhibitor of DNA binding 2
IDO: indoleamine 2,3-dioxygenase
ieILC1: Intraepithelial type 1 innate-like cell
IFN-γ: Interferon γ
IL: Interleukin
ILC1s: Type 1 innate-like cells
iNKT: Invariant natural killer T cell
iPSC: induced pluripotent stem cells
ITAM: Immunoreceptor tyrosine-based activation motif
ITIMs: Immunoreceptor tyrosine-based inhibitory motifs
KIRs: Killer cell immunoglobulin-like receptors
KLRs: Killer lectin-like receptors
LAG3: Lymphocyte-activation gene-3
LFA-1: Lymphocyte function-associated antigen-1
LILRs: Leukocyte immunoglobulin-like receptors
MCMV: Murine cytomegalovirus
MDSC: Myeloid-derived suppressor cell
MHC-I: Major histocompatibility complex-I
MIC: MHC I chain related molecule
MICA: MHC class I polypeptide-related sequence A
MICB: MHC class I polypeptide-related sequence B
NCRs: Natural cytotoxicity receptors
Nfil3: Nuclear factor interleukin-3-regulated protein
NK cell: Natural killer cell
NKG2: Nature-killer group 2
NKh: Helper natural killer
NKm: Memory natural killer
NKp30/44/46: Natural cytotoxicity receptor 30/44/46
NKreg: Regulatory natural killer
PBMCs: Peripheral blood mononuclear cells
pCR: Pathologic complete response
PD-1: Programmed cell death protein 1
PD-L1: Programmed cell death-ligand 1
PI3Ks: Class IA phosphatidylinositol 3 kinases
rhIL-15: Recombinant human interleukin − 15
scFv: Single-chain variable fragment
SREBP: Sterol regulatory element-binding proteins
T-bet: T-box transcription factor 21
TCRs: T cell receptors
TGF-β: Transforming growth factor-β
TIGIT: T-cell immunoglobulins and ITIM domain
TLR: Toll-like receptor
TME: Tumor microenvironment
TNF-α: Tumor necrosis factor α
Tox: Thymocyte selection associated high mobility group box
TriKEs: Trispecific killer engager
trNK cell: Tissue-resident natural killer cell
